# Iron Deficiency and Renal Phosphate Handling: The Role of Maximal Tubular Reabsorption of Phosphate Normalized to Glomerular Filtration Rate (TmP/GFR) in Proximal Tubular Dysfunction

**DOI:** 10.7759/cureus.76329

**Published:** 2024-12-24

**Authors:** Aman Yadav, Upma Narain, Arvind Gupta, Santosh Maurya

**Affiliations:** 1 General Medicine, Moti Lal Nehru Medical College, Prayagraj, IND; 2 Microbiology, Tejas Micro Diagnostic Centre, Prayagraj, IND; 3 Nephrology, Moti Lal Nehru Medical College, Prayagraj, IND

**Keywords:** chronic kidney disease, ferritin, iron deficiency, proximal tubular dysfunction, total iron-binding capacity

## Abstract

Background and aim: Phosphate dysregulation is often associated with chronic kidney disease (CKD), and recent studies suggest that it may also be present in non-CKD patients with systemic conditions including iron deficiency anemia. This study aimed to evaluate the relationship between iron deficiency parameters (total iron-binding capacity {TIBC}, hemoglobin, and serum ferritin) and markers of proximal tubular dysfunction (the maximal tubular reabsorption of phosphate normalized to glomerular filtration rate {TmP/GFR} and tubular reabsorption of phosphate {TRP}) in non-CKD patients with iron deficiency anemia.

Methods: This was a hospital-based analytical cross-sectional study conducted in the outpatient department and/or inpatient wards of the Department of Internal Medicine, Swaroop Rani Nehru (SRN) Hospital associated with Moti Lal Nehru (MLN) Medical College, Prayagraj, Uttar Pradesh, India, between July 2023 and August 2024.

Results: This study analyzed 40 anemic patients without CKD, with a mean age of 33.9 years. Most participants (n=24, 60%) were aged 18-35 years, and the majority (n=27, 67.5%) were female. Peripheral smear analysis revealed that 72.5% (n=29) had microcytic hypochromic anemia. Hemoglobin levels averaged 7.7 g/dL, serum iron was 91.0 µg/dL, total iron-binding capacity (TIBC) was 316.3 µg/dL, and serum ferritin was 199.7 ng/mL. Phosphate handling was assessed with TmP/GFR and tubular reabsorption of phosphate (TRP) showing mean values of 4.1 mg/dL and 99.2%, respectively. This study found that TmP/GFR had a significant positive correlation with TIBC (r=0.402, p=0.010), but non-significant negative correlations with hemoglobin and serum iron. TRP was negatively correlated with hemoglobin and serum ferritin, but not significantly. Among patients with microcytic hypochromic anemia, 55.2% (n=16) had increased TmP/GFR, and 61.1% (n=20) of patients with iron deficiency anemia exhibited increased TmP/GFR. Regression analysis revealed that TIBC significantly predicted TmP/GFR (p=0.022), indicating that higher TIBC values are associated with increased TmP/GFR, suggesting a potential link between iron metabolism and renal phosphate handling.

Conclusion: Higher TIBC levels were associated with increased TmP/GFR, suggesting that iron deficiency anemia may influence proximal tubular function. The findings emphasize the importance of considering renal phosphate handling in patients with iron deficiency anemia.

## Introduction

Iron deficiency anemia (IDA) is one of the most common nutritional disorders globally, affecting approximately 1.2 billion people [1,2]. While the systemic effects of IDA are well-documented, its potential impact on renal function, particularly proximal tubular function, has garnered increasing attention in recent years [3]. The proximal tubule of the nephron is a key site for the reabsorption of vital substances such as glucose, amino acids, and phosphate [4]. Phosphate homeostasis, in particular, is critical for maintaining bone health, cellular energy production, and overall metabolic balance [5]. Phosphate handling by the kidney is often assessed through measurements of tubular reabsorption of phosphate (TRP) and maximal tubular reabsorption of phosphate normalized to glomerular filtration rate (TmP/GFR) [6]. Altered phosphate handling, as indicated by reductions in TRP or TmP/GFR, has been implicated in a variety of clinical conditions, including chronic kidney disease (CKD) and metabolic bone disorders [7].

Although phosphate dysregulation is often associated with CKD, recent studies suggest that it may also be present in non-CKD patients with systemic conditions such as IDA [8,9]. Malyszko et al. found a moderate positive correlation between serum ferritin levels and proximal tubular dysfunction, raising the possibility that iron deficiency could impair renal tubular function [10]. This is biologically plausible, as iron plays a crucial role in cellular metabolism and oxidative stress regulation, both of which are vital for proper renal function [11]. Additionally, the altered oxygen-carrying capacity in IDA may lead to tissue hypoxia, further compromising kidney function [12]. Given the prevalence of IDA and the critical role of the kidney in maintaining homeostasis, it is important to explore the potential relationship between iron deficiency and renal tubular function in non-CKD patients. Against this background, the aim of the present study was to evaluate the relationship between iron deficiency parameters (TIBC, hemoglobin, and serum ferritin) and markers of proximal tubular dysfunction (TmP/GFR and TRP) in non-CKD patients with iron deficiency anemia.

## Materials and methods

Study design

This was a hospital-based analytical cross-sectional study conducted in the outpatient department and/or inpatient wards of the Department of Internal Medicine, Swaroop Rani Nehru (SRN) Hospital associated with Moti Lal Nehru (MLN) Medical College, Prayagraj, Uttar Pradesh, India, between July 2023 and August 2024.

Inclusion criteria

All patients more than 18 years of age, of both genders, presenting with anemia (based on hemoglobin levels with age and gender-specific cutoffs) were included in the present study. However, patients with known renal dysfunction with comorbidities, patients on drugs that can cause hemolytic anemia, those on drugs that can cause proximal tubular dysfunction, patients with thalassemia, and patients with hemolytic anemia were excluded from this study.

Sample size and sampling technique

Malyszko et al. conducted a research study and noted that there exists a significant positive moderate correlation (r=0.44; p<0.05) between levels of ferritin and proximal tubular dysfunction [10]. Based on this information, with an alpha error of 5% (type I error) and a beta error of 20% (type II error, or 80% power), the minimum estimated sample size was rounded to 40 patients with 95% confidence. We used a non-probability sampling technique, specifically complete enumeration, to enroll the patients.

Study procedure

A purpose-predesigned, semi-structured, pretested proforma was used to document the patient's sociodemographic characteristics (including age and gender), detailed clinical history, presence or absence of comorbidities, details of general physical examination, and clinical examination. It also included laboratory investigations, such as hemoglobin levels, peripheral smear, serum iron, total iron binding capacity, and serum ferritin. The proximal tubular dysfunction was assessed using tubular reabsorption of phosphate (TRP) and the maximal tubular reabsorption of phosphate normalized to glomerular filtration rate (TmP/GFR). The TRP formula mentioned below reflects the kidney's ability to reabsorb phosphate in the proximal tubule [13,14].

\[\text{TRP} = 1 - \left(\frac{U_p}{P_p} \times \frac{P_{cr}}{U_{cr}}\right)\]

By analyzing the ratio of urinary phosphate to plasma phosphate (Up/Pp) and comparing it to the ratio of plasma creatinine to urinary creatinine (Pcr/Ucr), TRP gives an estimate of how much phosphate is being reabsorbed versus excreted. A lower TRP might indicate impaired proximal tubular function, where phosphate reabsorption is compromised. TmP/GFR formula mentioned below helps to assess the kidney's maximum capacity to reabsorb phosphate, adjusted for the glomerular filtration rate (GFR) [13,14].

\[\text{TmP/GFR} = P_p - \left(\frac{U_p}{U_{cr}} \times P_{cr}\right)\]

It represents the point at which the proximal tubule becomes saturated with phosphate, beyond which any additional phosphate is excreted. TmP/GFR is crucial in understanding phosphate handling by the kidneys, especially in conditions that might lead to tubular dysfunction.

Statistical analysis

The data obtained were analyzed using SPSS version 23. All the categorical variables were summarized using frequencies and percentages. Continuous variables were summarized using mean (standard deviation). The correlation between levels of hemoglobin, serum iron, TIBC, and serum ferritin with levels of TRP and TmP/GFR were assessed using Pearson’s correlation coefficient. Statistical significance was considered at a p-value less than 0.05.

Ethical consideration

This study was approved by the Institutional Human Ethics Committee (IHEC), Moti Lal Nehru (MLN) Medical College, Prayagraj. The participants were given the Participant Information Sheet (PIS) in their native language, and its contents were verbally explained to ensure their understanding and satisfaction. enrolment in the study proceeded upon receipt of written informed consent.

## Results

The present study included a total of 40 patients with anemia and without chronic kidney disease. The mean age of the participants was 33.9 years, with a standard deviation of 13.6 years. The majority of the participants (n = 24, 60%) were between 18 and 35 years of age, followed by 22.5% (n = 9) in the 36-50 years age group, and 17.5% (n = 7) aged between 51 and 65 years. In terms of gender distribution, 32.5% (n = 13) of the patients were male, and 67.5% (n = 27) were female. The peripheral smear findings revealed that 72.5% (n = 29) of the patients had microcytic hypochromic anemia, while 10% (n = 4) had dimorphic anemia, and another 10% (n = 4) presented with a combination of microcytic hypochromic and macrocytic anemia. Normocytic normochromic anemia was observed in 7.5% (n = 3) of the patients, and none of the patients had macrocytic hypochromic anemia (Table 1).

**Table 1:** Distribution of patients by sociodemographic characteristics and peripheral smear findings. SD: standard deviation

Variables		Number (total = 40)	Percentage
Age (years)	18-35	24	60.0
	36-50	9	22.5
	51-65	7	17.5
	Mean (SD)	33.9 (13.6)	
Gender	Male	13	32.5
	Female	27	67.5
Peripheral smear	Dimorphic anemia	4	10.0
	Macrocytic hypochromic	0	0.0
	Microcytic hypochromic and macrocytic	4	10.0
	Microcytic hypochromic	29	72.5
	Normocytic normochromic	3	7.5

The study reported a mean hemoglobin level of 7.7 g/dL, with a standard deviation of 2.1. The mean serum iron level was 91.0 µg/dL, with a standard deviation of 62.0 µg/dL. The total iron binding capacity had a mean value of 316.3 µg/dL and a standard deviation of 73.2 µg/dL. Serum ferritin levels averaged 199.7 ng/mL, with a standard deviation of 182.0 ng/mL. Additionally, the mean TmP/GFR was 4.1 mg/dL, with a standard deviation of 2.2, and the mean tubular reabsorption of phosphate (TRP) was 99.2%, with a standard deviation of 0.6% (Table 2).

**Table 2:** Distribution of patients by parameters of iron and proximal tubular function. SD: standard deviation; TmP/GFR: the maximal tubular reabsorption of phosphate normalized to glomerular filtration rate; TRP: tubular reabsorption of phosphate

Variables	Mean	SD
Hemoglobin (g/dL)	7.7	2.1
Serum iron (µg/dL)	91.0	62.0
Total iron binding capacity (µg/dL)	316.3	73.2
Serum ferritin (ng/mL)	199.7	182.0
Tmp/GFR (mg/dL)	4.1	2.2
TRP (%)	99.2	0.6

Correlation between iron parameters, TmP/GFR, and TRP

The correlation analysis between TmP/GFR and various parameters showed a negative correlation with hemoglobin (r = -0.132, p = 0.416) and serum iron (r = -0.105, p = 0.518), although these correlations were not statistically significant. A positive and significant moderate correlation was observed between TmP/GFR and total iron binding capacity (r = 0.402, p = 0.010). Serum ferritin showed a negative but non-significant correlation with TmP/GFR (r = -0.099, p = 0.545) (Table 3).

**Table 3:** Correlation between iron parameters, TmP/GFR, and TRP. *P < 0.05 was considered statistically significant. SD: standard deviation; TmP/GFR: the maximal tubular reabsorption of phosphate normalized to glomerular filtration rate; TRP: tubular reabsorption of phosphate

Variables	Correlation coefficient (r)	p-Value
TmP/GFR		
Hemoglobin (g/dL)	-0.132	0.416
Serum iron (µg/dL)	-0.105	0.518
Total iron binding capacity (µg/dL)	0.402	0.010*
Serum ferritin (ng/mL)	-0.099	0.545
TRP		
Hemoglobin (g/dL)	-0.167	0.304
Serum iron (µg/dL)	0.047	0.772
Total iron binding capacity (µg/dL)	0.173	0.285
Serum ferritin (ng/mL)	-0.041	0.802

**Figure 1:**
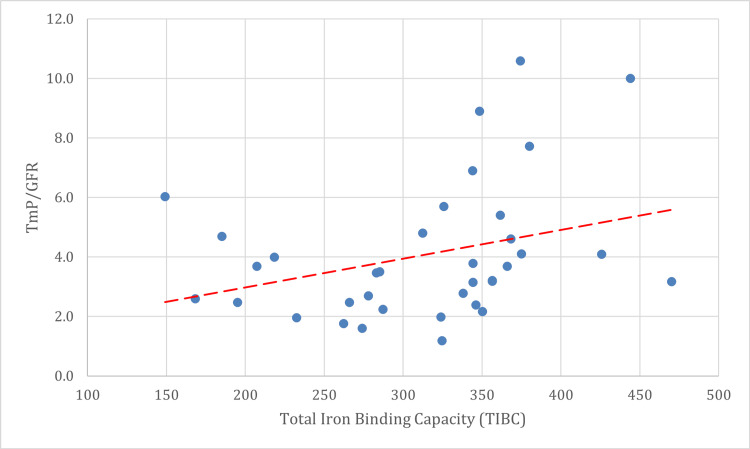
Correaltion between TIBC and TmP/GFR. TIBC: total iron-binding capacity; TmP/GFR: the maximal tubular reabsorption of phosphate normalized to glomerular filtration rate

For TRP, there was a negative correlation between hemoglobin (r = -0.167, p = 0.304) and serum ferritin (r = -0.041, p = 0.802), neither of which was statistically significant. There was a positive correlation between TRP and serum iron (r = 0.047, p = 0.772) and between TRP and total iron binding capacity (r = 0.173, p = 0.285), though both correlations were non-significant (Figure 1).

The results showed that of the 29 patients with microcytic hypochromic blood picture (72.5%), 16 patients (55.2%) had increased TmP/GFR. Also, it was noted that of the 18 patients with iron deficiency anemia (45.0%), all 18 had increased TIBC (100%), and 11 patients (11/18 patients, 61.1%) had increased TmP/GFR values. The regression analysis predicting TmP/GFR yielded statistically significant findings for TIBC. Specifically, TIBC was a significant predictor of TmP/GFR, with a regression coefficient (B) of 0.010, a standard error (SE) of 0.004, and a standardized beta coefficient (β) of 0.345. The t-statistic for this relationship was 2.391, and the corresponding p-value was 0.022, indicating that the association between TIBC and TmP/GFR is statistically significant. These results suggest that TIBC plays a meaningful role in predicting TmP/GFR, with higher TIBC values being associated with increases in TmP/GFR.

Correlation between phosphate parameters, TmP/GFR, and TRP

The correlation between phosphate parameters, TmP/GFR, and TRP reveals significant associations with plasma phosphate levels. TmP/GFR shows a perfect positive correlation with plasma phosphate (r = 1.000, p < 0.001), indicating a highly significant relationship (Table 4).

**Table 4:** Correlation between phosphate parameters, TmP/GFR, and TRP. *P < 0.05 was considered statistically significant. SD: standard deviation; TmP/GFR: the maximal tubular reabsorption of phosphate normalized to glomerular filtration rate; TRP: tubular reabsorption of phosphate

Variables	Correlation coefficient (r)	p-Value
TmP/GFR		
Plasma phosphate (mg/dL)	1.000	<0.001*
Urine phosphate (mg/dL)	-0.147	0.385
TRP		
Plasma phosphate (mg/dL)	0.538	0.001*
Urine phosphate (mg/dL)	-0.228	0.174

**Figure 2:**
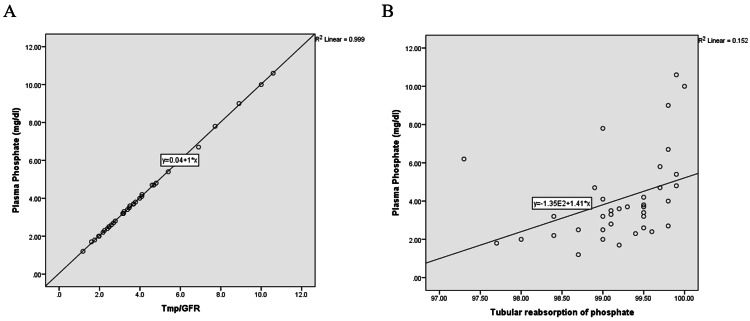
Correlation between phosphate parameters, TmP/GFR, and TRP. (A) Correlation between plasma phosphate and TMP/GFR; (B) correlation between plasma phosphate and TRP. TmP/GFR: the maximal tubular reabsorption of phosphate normalized to glomerular filtration rate; TRP: tubular reabsorption of phosphate

However, no significant correlation is observed between TmP/GFR and urine phosphate (r = -0.147, p = 0.385). Similarly, TRP exhibits a moderate positive correlation with plasma phosphate (r = 0.538, p = 0.001), which is statistically significant. In contrast, TRP's correlation with urine phosphate is weak and not significant (r = -0.228, p = 0.174) (Figures 2A, 2B).

## Discussion

The present study included a total of 40 patients with anemia and no chronic kidney disease (CKD), and it primarily aimed to determine the association between iron deficiency and proximal tubular dysfunction. The mean age of the patients was 33.9 years, with a predominance of patients in the age group of 18-35 years (60%). This finding aligns with global reports showing a higher prevalence of anemia among young adults, particularly in reproductive-aged women [15,16]. The predominance of female participants (67.5%) reflects the well-known gender disparity in anemia prevalence, driven largely by factors such as menstruation, pregnancy, and malnutrition [17]. In terms of peripheral smear findings, the majority of participants (72.5%) exhibited microcytic hypochromic anemia, typically indicative of iron deficiency. Microcytic hypochromic anemia results from reduced hemoglobin synthesis, often associated with iron deficiency, thalassemia, or chronic diseases [18,19]. Given that none of the patients had CKD, the findings suggest iron deficiency as the leading cause of anemia, which is consistent with global trends [20].

The mean hemoglobin level of 7.7 g/dL indicates moderate anemia according to the World Health Organization (WHO) classification [21]. Low hemoglobin levels are strongly associated with iron deficiency, which is reflected in the serum iron and ferritin levels observed in the study population. The mean serum iron level of 91.0 µg/dL was slightly lower than the normal range, but the serum ferritin level of 199.7 ng/mL appears elevated. This could be explained by the presence of anemia of chronic disease in a subset of participants, as ferritin levels may increase in response to inflammation despite concurrent iron deficiency [22,23]. Elevated total iron-binding capacity (TIBC) with low serum iron is another hallmark of iron deficiency, and the mean TIBC of 316.3 µg/dL supports this diagnosis [24,25].

The study's primary objective was to assess proximal tubular function using two key parameters as follows: TRP and TmP/GFR. The mean TRP of 99.2% suggests that proximal tubular reabsorption of phosphate was almost complete in the majority of participants. High TRP values have been associated with conditions such as hypophosphatemia and may indicate a compensatory mechanism in response to low serum phosphate levels [26,27]. The mean TmP/GFR of 4.1 mg/dL observed in the present study is consistent with normal phosphate handling by the kidneys. TmP/GFR is a critical measure of the kidney's ability to reabsorb phosphate, and its values may be influenced by factors such as iron status. Previous studies have suggested that iron deficiency can impair renal phosphate reabsorption due to alterations in fibroblast growth factor-23 (FGF23) levels, a key regulator of phosphate homeostasis [28]. Specifically, Malyszko et al. demonstrated a significant correlation between ferritin levels and proximal tubular dysfunction, with a moderate positive correlation (r = 0.44; p < 0.05) [10]. In our study, the elevated ferritin levels observed in some participants may be linked to disruptions in phosphate handling, possibly via FGF23 dysregulation [29]. Iron deficiency has been implicated in the development of both renal phosphate wasting and renal tubular acidosis (RTC), conditions characterized by proximal tubular dysfunction [30]. Furthermore, iron supplementation in patients with iron deficiency anemia has been shown to improve phosphate reabsorption and restore normal TRP and TmP/GFR values [28].

The negative correlations observed between TmP/GFR and both hemoglobin (r = -0.132, p = 0.416) and serum iron (r = -0.105, p = 0.518), although not statistically significant, suggest a potential inverse relationship between iron status and proximal tubular phosphate handling [31]. This observation may point to a complex interplay between iron deficiency and renal phosphate reabsorption mechanisms [6]. Prior studies have indicated that iron deficiency may alter phosphate metabolism through its effects on fibroblast growth factor 23 (FGF23), a hormone that regulates phosphate homeostasis and is known to be elevated in iron-deficient states [28]. However, the lack of statistical significance in the present study might be attributed to the relatively small sample size, which could limit the power to detect subtle correlations. A significant positive moderate correlation was found between TmP/GFR and TIBC (r = 0.402, p = 0.010), which indicates that as TIBC increases, TmP/GFR also tends to increase. TIBC is often elevated in iron deficiency, reflecting the body's increased capacity to bind iron due to lower circulating iron levels [32,33]. The positive correlation between TmP/GFR and TIBC could be indicative of compensatory mechanisms in response to iron deficiency, whereby the kidneys attempt to preserve phosphate by increasing its reabsorption. This finding aligns with prior evidence suggesting that iron deficiency may lead to alterations in proximal tubular function, potentially via increased FGF23 activity [28]. The regression analysis provided further insights into the relationship between iron deficiency and proximal tubular function. TIBC emerged as a significant predictor of TmP/GFR, with a regression coefficient of 0.010 (SE = 0.004) and a standardized beta coefficient (β) of 0.345. The association between TIBC and TmP/GFR was statistically significant, with a t-statistic of 2.391 and a p-value of 0.022. These results suggest that higher TIBC values are associated with increased TmP/GFR, indicating a compensatory increase in phosphate reabsorption in response to iron deficiency. Serum ferritin showed a negative but non-significant correlation with TmP/GFR (r = -0.099, p = 0.545). Elevated serum ferritin is typically associated with inflammation or iron overload, whereas low ferritin levels are indicative of iron deficiency [18]. The inverse relationship observed in this study could suggest that lower ferritin levels are associated with impaired phosphate reabsorption, possibly due to decreased availability of iron, which is necessary for optimal renal function [34]. However, the lack of significance underscores the need for further research to elucidate the potential mechanisms underlying this relationship.

The correlation analysis for TRP revealed a pattern similar to that of TmP/GFR, with negative correlations between TRP and hemoglobin (r = -0.167, p = 0.304) and between TRP and serum ferritin (r = -0.041, p = 0.802). Although these correlations were not statistically significant, they are consistent with findings from other studies that suggest iron deficiency may negatively affect renal tubular phosphate reabsorption [35]. In contrast, positive correlations were observed between TRP and serum iron (r = 0.047, p = 0.772) and between TRP and TIBC (r = 0.173, p = 0.285), though neither of these correlations was statistically significant. The positive correlation between TRP and serum iron suggests that higher serum iron levels may support better phosphate reabsorption in the proximal tubules, possibly due to improved tubular function in the presence of adequate iron stores. The findings of the present study suggest that iron deficiency may be associated with alterations in proximal tubular phosphate handling, as evidenced by the correlations between TmP/GFR, TRP, and iron parameters.

Among the 29 patients with a microcytic hypochromic blood picture (72.5%), more than half (55.2%) had elevated TmP/GFR values, suggesting that altered phosphate handling may be prevalent in individuals with this anemia subtype. Microcytic hypochromic anemia is typically a hallmark of iron deficiency, which may explain the observed trends in phosphate reabsorption. Furthermore, all 18 patients with iron deficiency anemia (45.0%) had increased TIBC, and a majority (61.1%) had elevated TmP/GFR, reinforcing the notion that iron deficiency may significantly impact phosphate reabsorption in the proximal tubules. These findings are consistent with previous research showing that iron deficiency can lead to disruptions in mineral metabolism, particularly phosphate homeostasis [28].

Higher TmP/GFR and TRP both indicate increased renal phosphate retention. In physiological terms, when the proximal tubules of the kidneys increase their capacity to reabsorb phosphate (high TmP/GFR) and efficiently reabsorb a greater proportion of filtered phosphate (high TRP), less phosphate is excreted in the urine, and more is returned to the bloodstream. Briefly, as more phosphate is reabsorbed and retained, plasma phosphate rises.

The present study, while providing valuable insights into the association between iron deficiency anemia and renal phosphate handling, is subject to several limitations as follows: it includes single-center study design limits the external validity of the findings, cross-sectional nature of the study limits the ability to establish causal relationships, limited control of confounding variables (such as nutritional status, hormonal imbalances (e.g., parathyroid hormone, vitamin D), or medications that affect phosphate metabolism), limited biomarkers being considered (other potential markers could be serum phosphate levels, urinary phosphate excretion, or FGF23), absence of comparative group, and potential measurement bias.

## Conclusions

The present study highlights a significant association between iron deficiency anemia and altered phosphate handling in non-CKD patients, as evidenced by the observed relationship between TIBC and maximal tubular reabsorption of phosphate normalized to glomerular filtration rate. Specifically, higher TIBC levels were associated with increased TmP/GFR, suggesting that iron deficiency anemia influences proximal tubular function and increases phosphate absorption in the proximal convoluted tubule.

This study underscores the importance of anemia, specifically iron deficiency anemia in the alteration of proximal tubular function, which can have effects on normal physiology and gives us future insights to look upon, as an uncorrected state could lead to progressive decline in renal tubular function and finally into CKD. This study adds to the knowledge of health policymakers and healthcare providers that not only can CKD be a cause of anemia, but anemia can also lead to renal tubular dysfunction. By preventing and correcting anemia early, these complications may be managed.
